# ADATscan – A flexible tool for scanning exomes for wobble inosine-dependent codons reveals a neurological bias for genes enriched in such codons in humans and mice

**DOI:** 10.17912/micropub.biology.000675

**Published:** 2023-01-16

**Authors:** Emery R. Longan, Jillian Ramos, Dragony Fu

**Affiliations:** 1 University of Rochester, Department of Biology, Rochester, NY 14620; 2 Department of Biochemistry and Molecular Genetics, University of Colorado Anschutz Medical Campus, School of Medicine, Aurora, CO 80045

## Abstract

The conversion of adenosine to inosine at the wobble position of select tRNAs is essential for decoding specific codons in bacteria and eukarya. In eukarya, wobble inosine modification is catalyzed by the heterodimeric ADAT complex containing ADAT2 and ADAT3. Human individuals homozygous for loss of function variants in ADAT3 exhibit intellectual disability disorders. We created a flexible computational tool to scan the human, mouse, nematode, fruit fly, and yeast exomes for genes either enriched or depleted in ADAT-dependent codons as compared to background models of codon bias derived from the exomes themselves. We find that many genes are enriched or depleted for ADAT-dependent codons as compared to the genomic background in all five species. Among those genes enriched for ADAT-dependent codons in humans, we find there is significant Gene Ontology (GO) enrichment for genes involved in diverse neurological processes. This pattern persists in the mouse exome but not the fruit fly or nematode exome. In the nematode exome, genes enriched in ADAT-dependent codons are GO enriched for translation associated genes, and in yeast there is GO enrichment for genes involved in metabolic functions. There is also GO-term overlap between yeast and fruit flies. Importantly, in its generalized form, ADATscan can also be used to scan any exome for genes enriched in any subset of codons specified by the user.

**Figure 1. Results derived from ADATscan:**
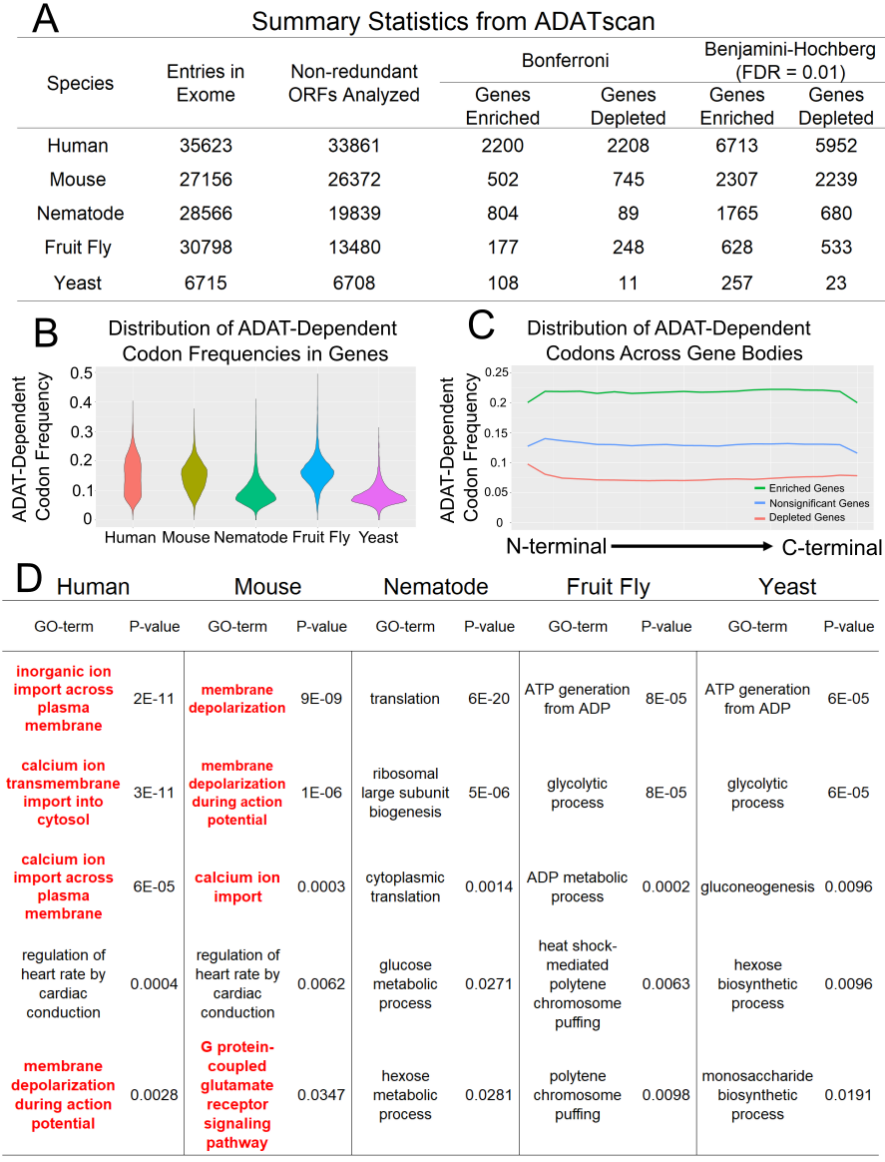
A) Summary statistics regarding the genes analyzed using ADATscan. B) The distribution of ADAT-dependent codon frequencies within genes in the human, mouse, nematode, fruit fly, and yeast exomes as determined by ADATscan. C) Average ADAT-dependent codon frequencies in enriched, depleted, and nonsignificant genes across gene bodies for all five species. D) GO-term enrichment using the list of genes that are enriched for ADAT-dependent codons after correcting for multiple comparisons (Benjamini-Hochberg procedure, FDR = 0.01). In cases where more than 500 genes were enriched, the 500 genes with the smallest p-values were used for GO-term analysis. The top five categories by fold enrichment output by PANTHER (see Reagents) are shown ordered by their p-values for each of the five species. Neurological associated processes are noted in bold and red.

## Description

Modification of wobble adenosine to inosine in the anticodon of tRNAs is a conserved enzymatic reaction, catalyzed by the TadA homodimeric complex in *E. coli* (Wolf et al. 2002) or the ADAT2/3 heterodimeric complex in eukaryotes (Gerber and Keller 1999; Rubio et al. 2007; Torres et al. 2015). Wobble inosine modification by ADAT2/3 is indispensable in eukaryotes for decoding certain codons, though the subsets are slightly variable depending on the specific eukaryotic organism (Percudani et al. 1997; Rafels-Ybern et al. 2015). Human individuals homozygous for loss of function mutations in ADAT3 exhibit intellectual disability disorder (Alazami et al. 2013; El-Hattab et al. 2016), and in extreme cases, microcephaly (Sharkia et al. 2019). Previous studies have identified specific molecular defects associated with pathogenic ADAT3 variants that cause intellectual disability (Ramos and Fu 2019; Ramos et al. 2020; Ramos-Morales et al. 2021). However, the precise causes for human cognitive disorders caused by wobble inosine deficiency remain elusive.

To screen for genes that may be the most (or the least) likely to be translationally impacted by decreased ADAT activity, we created a computational tool to scan exome data for enrichment or depletion of ADAT-dependent codons. Our methodology is to first establish a background model for ADAT-dependent codon usage in the exome (see Methods). This null model is then applied to each nonredundant gene entry in an exome FASTA file whereby an expected number of ADAT-dependent codons is calculated and compared to the observed number via simple chi-square test (Figure 1A). These chi-square tests are then corrected for multiple comparisons (Benjamini-Hochberg procedure, FDR = 0.01) to yield a conservative list of genes that are enriched or depleted for ADAT-dependent codons. This tool also outputs the frequency of ADAT-dependent codons in each gene (Figure 1B), the counts of these codons in each gene, and the p-values for enrichment or depletion of ADAT-dependent codons for each gene. The tool also outputs a file that allows for plotting ADAT-dependent codon frequencies across gene bodies (Figure 1C).

Using this tool, we identified 5952 human genes that are significantly depleted for ADAT-dependent codons and 6713 human genes that are significantly enriched for ADAT-dependent codons as compared to the human exomic background. Subsequent GO-term enrichment analysis showed that genes enriched in ADAT-dependent codons are biased for genes involved in neurological associated processes (Figure 1D). The human genes identified represent candidates that may underlie the neurodevelopmental phenotypes seen in patients homozygous for loss of function variants in ADAT3. Notably, mouse genes enriched for ADAT-dependent codons also exhibit an overrepresentation of genes involved in neurological processes (Figure 1D). In contrast, no enrichment for genes involved in neurological processes was found within the fruit fly or nematode exome (Figure 1D). The nematode exome showed GO-term enrichment among those genes enriched for ADAT-dependent codons for translation associated genes. For the yeast exome, genes enriched for ADAT-dependent codons are GO-term enriched for genes involved in metabolic functions, and there is some GO-term overlap between fruit fly and yeast (Figure 1D). There was no striking trend in terms of ADAT-dependent codon frequency across gene bodies among enriched, depleted, and nonsignificant genes (Figure 1C). Altogether, these studies demonstrate that ADATscan can be used to predict which genes are presumably most dependent on ADAT for efficient translation and serve as a starting point for identifying biological processes linked to wobble inosine modification. Importantly, ADATscan is user-friendly and can be applied to any exome for any set of codons based on user input.

## Methods

*Definition of ADAT-dependent codon*

The definition of an ADAT-dependent codon is a -C or -A ending codon which lacks a cognate tRNA for decoding it, and is thus dependent on wobble inosine modified tRNAs. This information can be found at http://gtrnadb.ucsc.edu/ (Data Release 19). For humans, mice, nematodes, and fruit flies this codon set is: ACC, GCC, CCC, TCC, CTC, ATC, GTC, CGC. For yeast, the codon set is: TCC, CCC, CGC, CGA, ATC, ACC, GTC, GCC. Of note, humans, unlike most other eukaryotes, have three cognate tRNAs to decode ATC. However, these tRNAs are lowly expressed (Cozen et al. 2015). As such, this codon was retained in the analysis for humans.

*Background model*

The background model for ADAT-dependent codon usage is calculated using the relevant set of codons and the relevant exome data. Every codon in the exome is translated. If a codon corresponds to an amino acid that is encoded by an ADAT-dependent codon, then it is counted and recorded. This is done separately for all relevant amino acids. For each of these amino acids, the number of instances of ADAT-dependent codons are also counted. The latter count is divided by the former count for each relevant amino acid, yielding the background frequency of ADAT-dependent codon usage for each amino acid. This model is output to a file by ADATscan.

*Testing for enrichment and depletion*

For each sequence in the exome file, amino acids that are encoded by ADAT-dependent codons are counted separately. These counts are then multiplied by their respective background frequency of ADAT-dependent codon usage in the exome as calculated in the background model. These numbers are then summed to yield a null estimate for the number of ADAT-dependent codons expected to appear in the protein based on amino acid composition and the background frequency of ADAT-dependent codon usage. This expectation is then compared to the observed number of ADAT-dependent codons in a 2x2 chi-square table that is filled as shown below:

**Table:** 

	Observed	Expected
Protein length	Empirical value	Empirical value
ADAT-dependent codons	Empirical value	Expected value based on background model

These tests are then corrected for multiple comparisons (Benjamini-Hochberg procedure, FDR = 0.01) to yield a conservative list of genes that are enriched or depleted for ADAT-dependent codons. In cases where more than 500 genes were enriched, the 500 genes with the smallest p-values were used for GO-term enrichment.

## Reagents

Human and mouse exome data were retrieved from the CCDS database ( https://ftp.ncbi.nlm.nih.gov/pub/CCDS/ , current release: 12/11/22). CCDS IDs were converted to gene names for GO-term analysis using g:Profiler ( https://biit.cs.ut.ee/gprofiler/convert , Version: e107_eg54_p17_bf42210). The nematode exome data were retrieved from WormBase ( https://downloads.wormbase.org/releases/WS287/species/c_elegans/PRJNA13758/ , release: WS287). Fruit fly exome data were retrieved from FlyBase ( http://ftp.flybase.net/genomes/Drosophila_melanogaster/current/fasta/ , release: FB2022_06). Yeast exome data were retrieved from the *Saccharomyces* Genome Database ( http://sgd-archive.yeastgenome.org/sequence/S288C_reference/orf_dna/ , current release: 4/22/2021). GO-term enrichment was performed using PANTHER (Thomas et al. 2022) for all species using a Fisher’s exact test and a Bonferroni correction ( http://geneontology.org/ , PANTHER release 17.0 , GO Ontology database DOI: 10.5281/zenodo.6799722). ADATscan and the outputs obtained in this study are freely available and can be found at https://github.com/elonganrochester/ADATscan . Five versions are provided. One tailored to ADAT-dependent codon detection in CCDS, one tailored to ADAT-dependent codon detection in nematodes, one tailored to the files from Flybase, one tailored to ADAT-dependent codon detection in yeast, and a generalized user-friendly version that can take any codon set and any exome FASTA file as input. All versions allow for user input regarding the desired multiple-testing correction as either the Bonferroni correction or the Benjamini-Hochberg procedure with a user-specified FDR. An example of how to run ADATscan is also provided.

## Extended Data

Description: This file contains the results of ADATscan when the Bonferroni correction is applied. Resource Type: Dataset. DOI: 10.22002/krvth-eb002

Description: This file contains the results of ADATscan when the Benjamini-Hochberg procedure is applied. Resource Type: Dataset. DOI: 10.22002/fs9vf-svg51

Description: Link to github. Resource Type: InteractiveResource. DOI: 10.22002/z1437-thb52
